# Standardized Measurement of Implementation: The Universal SIC

**DOI:** 10.1186/1748-5908-10-S1-A73

**Published:** 2015-08-20

**Authors:** Lisa Saldana, Holle Schaper, Mark Campbell, Jason Chapman

**Affiliations:** 1 Oregon Social Learning Center, Eugene, OR, 97401, USA

## Introduction

Although recent advances have improved understanding of what it takes to successfully implement and sustain evidence-based practices (EBPs) in communities and to transform health care systems, there remains a dearth of knowledge regarding methods for measuring and subsequently evaluating these efforts. Recognizing the complexity of the process, which involves planning, training, quality assurance, and interactions among multiple stakeholder groups, the Stages of Implementation Completion (SIC) was developed.

## Background and Methods

The SIC is an 8-stage tool that maps onto three phases of implementation (pre-implementation, implementation, and sustainability), and was developed as part of an implementation trial to assess sites' implementation process behavior and obtainment of milestones. Developed for an EBP for youth in the child welfare and juvenile justice systems, the SIC has demonstrated the ability to assess and predict meaningful implementation outcomes. Since that time, the SIC has been adapted for a number of EBPs across different service systems (e.g., school, health care) and at different points of system entry (e.g., prevention, intervention).

This presentation will describe recent efforts to combine these practice specific SIC measures to understand what implementation elements are common across different implementation strategies. Using these multiple adaptations, an empirically driven Universal SIC was developed. The multi-staged Universal SIC developmental process and the complexities that arise when defining and comparing implementation processes and outcomes across different implementation strategies will be described. The iterative coding and agreement process will be presented, and decision rules outlined. Preliminary findings will be presented comparing outcomes obtained when EBP implementations are analyzed using the Universal SIC versus the practice-specific SIC.

## Advancing the Field

Discussion will highlight the value of having a standardized measure to compare outcomes across practices, evaluations, and fields, yet will also describe the loss of sensitivity moving from practice-specific to universal measurement.

## Funding

R01MH097748; P50DA035763

